# Selective Intramolecular Dehydrocyclization of Co-Porphyrin on Au(111)

**DOI:** 10.3390/molecules25173766

**Published:** 2020-08-19

**Authors:** Cen Yin, Zhantao Peng, Dan Liu, Huanjun Song, Hao Zhu, Qiwei Chen, Kai Wu

**Affiliations:** BNLMS, College of Chemistry and Molecular Engineering, Peking University, Beijing 100871, China; 18811710898@163.com (C.Y.); pengzhantao@pku.edu.cn (Z.P.); frankliu0519@pku.edu.cn (D.L.); songhuanjun@pku.edu.cn (H.S.); 1701110426@pku.edu.cn (H.Z.)

**Keywords:** C–H activation, Co-porphyrin, on-surface reaction, scanning tunneling microscopy

## Abstract

The on-surface C–H bond activation and coupling reaction is a powerful approach to constructing fine-tuned surface nanostructures. It is quite challenging to control its regioselectivity due to the inertness of the C–H bond involved. With scanning tunneling microscopy/spectroscopy and theoretical calculations, the C–H activation and sequential intramolecular dehydrocyclization of *meso*-tetra(p-methoxyphenyl)porphyrinatocobalt(II) was explored on Au(111), showing that the methoxy groups in the molecule could kinetically mediate the selectivity of the intramolecular reaction over its intermolecular coupling counterpart. The experimental results demonstrate that the introduced protecting group could help augment the selectivity of such on-surface reaction, which can be applied to the precise fabrication of functional surface nanostructures.

## 1. Introduction

On-surface synthesis emerged in the last decade as an efficient approach to achieving novel chemical reactions/products and fabricating elaborate surface molecular nanostructures [1,2,3,4,5,6]. Among various types of on-surface reactions, the C–H bond activation and coupling (abbreviated as C–H BAC) reaction [7,8] has been one of the most concerned, not only due to its difficulty in traditional wet chemistry, but also for its convenience and atomic efficiency in fabricating surface nanostructures such as graphene nanoribbons [9,10], conjugated porphyrin wires [11] and two-dimensional covalent polymers [12,13].

Due to the inertness and ubiquity of C–H bonds in molecular precursors, the C–H BAC reaction on surface normally performs poorly in both reactivity and selectivity. New on-surface strategies such as surface confinement [14], self-assembly mediation [15], thermal control [16], halogen-adatom-promoted reactivity [17] and so on and so forth, have successfully been applied to enhance the reactivity and improve the regioselectivity in some specific cases. Recently, metalloporphyrin and other tetrapyrrole derivatives have been reported to undergo C–H BAC reactions, such as dehydrocyclization (DHC) [18,19,20,21], coupling reactions with graphene [21,22] and intermolecular dehydro-couplings [11,16,18,20]. Owing to the wide applications of porphyrin and its derivatives in molecular electronics [23,24] and catalysis [25,26], such studies have drawn intense interest. For instance, the *β*-H of the pyrrole ring or the *para*-H of the *meso*-phenyl group in these molecules can be activated at elevated temperatures and interlinked with adjacent groups, molecules and nano-entities on the surface. This leads to the fact that intramolecular and intermolecular couplings take place along different reaction paths, resulting in the regioselectivity of the C–H BAC reaction of porphyrins on the surface. Several reports have described the selective control of intra and inter-molecular products of porphyrins by thermal [16] and surface [27] effects. However, to our knowledge, few studies [10,20] have investigated the introduction of protecting groups to promote the selectivity of intra or inter-molecular reactions on the surface, possibly because the high C–H activation energy requires an elevated reaction temperature that few substituent groups could survive on the surface.

In this study, low temperature scanning tunneling microscopy (LT-STM) was employed to investigate on-surface reactions of *meso*-tetra(*p*-methoxyphenyl)porphyrinatocobalt(II) [Co(TAP)] (“A” standing for anisyl, an alias for methoxyphenyl) on Au(111). In Co(TAP), the *para*-positions of its four *meso*-phenyl groups were substituted with methoxy groups. As the bond strength is comparable between the C–O and C–H bonds, such methoxy groups are likely to survive high temperature treatment that are necessarily required to activate the C–H bonds. Therefore, the intermolecular coupling at the para-sites of *meso*-phenyl groups could be suppressed. Our STM measurements clearly identified the intramolecular DHC products of Co(TAP) on Au(111) at elevated temperatures. Combined scanning tunneling spectroscopy (STS) and theoretical calculations further supported the reaction products formed. Experimentally observed and resolved reaction intermediates indicated that methoxy substituents could stay intact at a temperature whereat the DHC reaction started. The peculiar observation of the intermolecular coupling products demonstrated the profound selectivity of the reaction based on statistical analyses.

## 2. Results and Discussion

### 2.1. Dehydrocyclization of Co(TAP) on Au(111)

**Figure 1:**
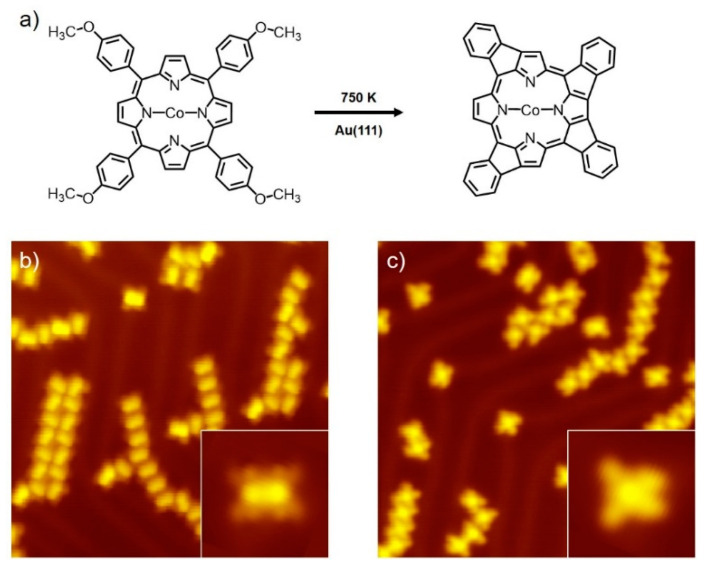
(**a**) Schematic illustration of the DHC reaction of Co(TAP) into the reaction product at 750 K on Au(111); (**b**) STM image of Co(TAP) adsorbed on Au(111) at room temperature and at low coverage; inset: enlarged STM image of an individual Co(TAP) molecule; (c) STM image of the DHC product generated from 750 K annealing of the sample in (**b**); inset: enlarged STM image of an individual DHC product. Imaging conditions for STM images were −0.3 V and 20–50 pA. Image size for (**b**,**c**): 30 × 30 nm^2^; size for inset images: 4 × 4 nm^2^.

The chemical structure of Co(TAP) is schematically depicted at the left side of Figure 1a. Upon its adsorption on the Au(111) surface, the Co(TAP) molecule adopts a saddle-like configuration [28]. The STM image of an individual Co(TAP) molecule (inset in Figure 1b) present several key features that well match the chemical structure of Co(TAP): (1) A bright rod-like protrusion at the molecule center represents two up-ward tilted pyrrole rings and the central Co atom. (2) Four dim spots at both sides of the bright rod are due to the *meso*-phenyl groups. (3) Claw-shaped protrusions around four dim spots stem from the methoxy groups [29]. At room temperature, low-coverage adsorption of the Co(TAP) molecules led to the formation of sparsely distributed self-assembled structures on the surface, in accordance with our previous study [28].

**Figure A3:**
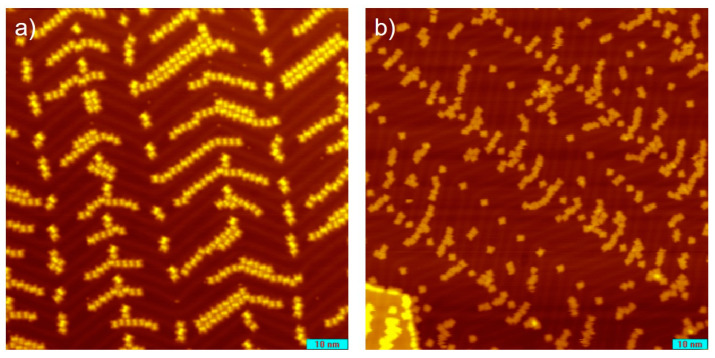
Large-scale STM images of Co(TAP) adsorbed Au(111) surface annealed (**a**) at room temperature, and (**b**) at 750 K. The blue scale bars in (**a**,**b**) represent 10 nm. Imaging conditions: (**a**) −1.0 V, 30 pA; (**b**) 2.5 V, 60 pA.

After being annealed at about 750 K for 2 min, the surface molecular coverage did not decrease distinctly. Figure 1b,c and Figure A3 show the general appearance of the sample surface before and after the DHC reaction. However, most molecules exhibited an imaged feature different from that of Co(TAP) molecule deposited at room temperature. A typical STM image of such species is shown in the inset of Figure 1c. In contrast to untreated Co(TAP), different regions in the thermally treated molecule possess a similar contrast in the image, suggesting that the thermally treated molecule adopted a planar adsorption configuration. Besides, although the thermally treated molecule shows a four-lobed feature similar to that for untreated Co(TAP), it is obvious that the angle formed by the left two lobes is larger than that by the right counterparts. Moreover, the claw-shaped protrusions were not observable, implying that the four methoxy groups dissociated. According to above-described experimental observations, the chemical structure of the treated molecule was proposed (right side of Figure 1a). It shows that the thermally treated molecule was actually a DHC product between the *β*-H in the pyrrole group and *ortho*-H in the *meso*-phenyl group. Similar products were previously reported for 5,15-diphenylporphyrin (2H-DPP) on Cu(111) [18], tetrakis-(4-fluorophenyl)porphyrin (2H-4FTPP) [20], Fe-tetra(4-bromophenyl)porphyrin chloride [Br_4_-FeTPP(Cl)] [21] on Au(111) and Zn-*β*-tetrabromo-tetraphenylporphyrin (ZnTPPBr_4_) in wet synthesis [30]. Due to the fact that *meso*-phenyl may react with the pyrrole group either on its left or its right side, there exist four structural isomers for the DHC product (Appendix A). Additionally, we note that the hydroxy group is normally unidentifiable by STM imaging. Consequently, the possibility of the methoxy group’s partial dissociation into a hydroxy group cannot be completely ruled out. Nevertheless, structural analysis on traces of intermolecular coupling products indicated that the DHC products were free of the hydroxyl species because no ether-type intermolecular coupling product was found (Appendix B).

**Figure 2:**
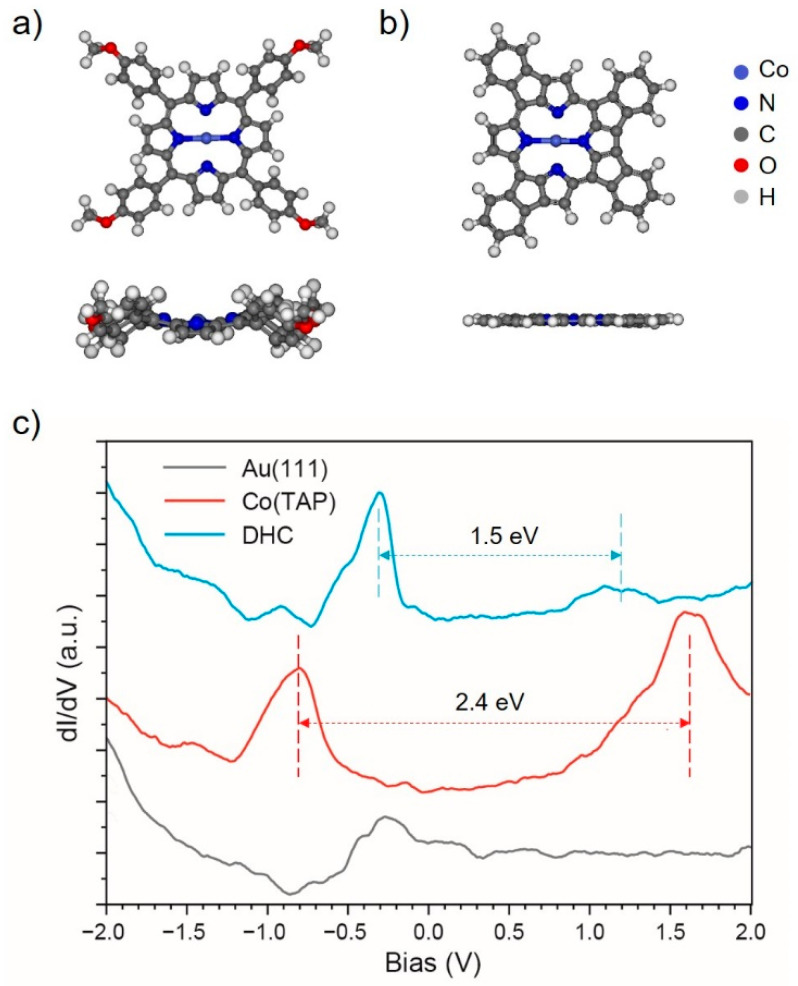
Bird and side views of the optimized structures of (**a**) Co(TAP) and (**b**) DHC products in free space; (**c**) STS spectra acquired on Au(111), Co(TAP) and DHC product.

STS and theoretical calculations further supported formation of the DHC products. Figure 2a,b show the optimized structures of Co(TAP) and DHC products in free space by density functional theory (DFT) calculations. A side view of the structures points out that the DHC product adopts a planar configuration while the Co(TAP) molecule adopts a saddle-like one. This explains the uniformity of image contrast for an individual DHC product observed in STM. The STS spectra were correspondingly acquired on Au(111) and at centers of the Co(TAP) and DHC products (Figure 2c). The surface-state-related spectral feature of the Au(111) substrate appears at about −0.3 V. For Co(TAP), two characteristic peaks are positioned at about −0.8 V and 1.6 V (marked by the red dashed lines), corresponding to the highest occupied molecular orbital (HOMO) and lowest unoccupied molecular orbital (LUMO) of the Co(TAP) molecule adsorbed on Au(111). The surface state of Au(111) is possibly buried in the tail of the −0.8 V peak and hence becomes indistinguishable in the spectrum. For the DHC product, the peak at −0.3 V is remarkably distinct from that for the Au(111) surface state in both peak shape and intensity, featuring the spectroscopic properties of the DHC product. Another small hump was observed at about 1.2 V. These spectroscopic peaks were identified as the HOMO and LUMO of the DHC product on Au(111), as marked by the blue dashed line. Compared with Co(TAP), the HOMO–LUMO gap of the DHC product narrows down from 2.4 to 1.5 eV. This is understandable by the fact that the DHC product is more conjugative than Co(TAP). The DFT calculations for the Co(TAP) molecule and DHC product in free space also support such a narrowing-down of the HOMO–LUMO gap, as DFT calculated HOMO–LUMO gaps were 2.9 eV and 2.1 eV for the Co(TAP) molecule and DHC product, respectively. The absolute values of calculated HOMO–LUMO gaps were different from those measured in STS. The reason is quite obvious: the DFT calculations were conducted in free space wherein the surface effect on the molecule was not included.

### 2.2. Intermediates of the Dehydrocyclization Reaction

**Figure 3:**
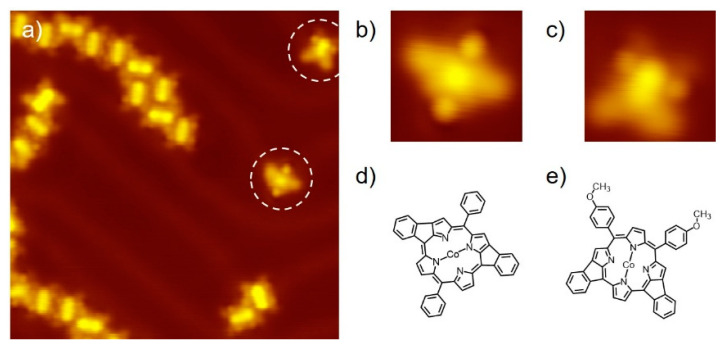
STM image of (**a**) annealed Co(TAP) self-assembly on surface at 523 K; (**b**,**c**) two types of intermediates as the dehydrocyclization products where only two of the four *meso*-methyl groups are coupled with the pyrrole groups; (**d**,**e**) proposed chemical structures for the products shown in (**b**,**c**), respectively. Imaging conditions: −0.3 V, 20 pA. Image size: (a) 20 × 20 nm^2^; (**b**,**c**) 4 × 4 nm^2^.

Once the surface was annealed at a medium temperature, i.e., 523 K, for 2 min, partially yielded DHC products were observed on the surface. As shown in Figure 3a, most Co(TAP) molecules remained intact and formed small domains of self-assembly structures. The methoxy groups presented as claw-shaped features can also be identified in the STM image. In addition, several molecules with novel morphologies were identified, as marked by the white dashed circles. Enlarged STM images (Figure 3b,c) indicate that such novel molecules are partially generated DHC products with two *meso*-phenyl groups coupled with their adjacent pyrrole groups. The chemical structures of these two intermediates are schematically proposed in Figure 3d,e, which well match the STM morphologies.

Another finding was that the methoxy groups in some partially produced DHC products survived, as the “claw” feature in STM image can be clearly observed (Figure 3c). This means that the dissociation of the methoxy group requires an activation energy higher than or at least nearly equivalent to that of the DHC reaction. Therefore, there is a chance that the methoxy groups serve as a protecting group to prevent the intermolecular dehydro-coupling reactions from happening at elevated temperatures.

### 2.3. Intra and Inter-Molecular Reaction Selectivity

**Figure 4:**
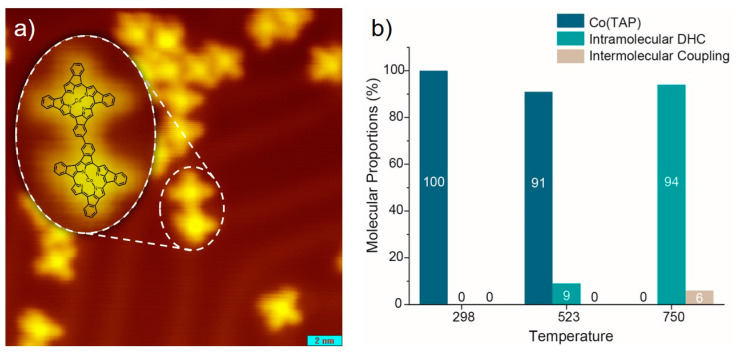
(**a**) An STM image of the annealed Co(TAP) self-assembly on the surface at 750 K. The intermolecular coupling product is marked by the white dashed oval and superimposed with its chemical structure on an enlarged image. (**b**) Statistic histograms of Co(TAP), intramolecular DHC and intermolecular coupling products at different reaction temperatures. Imaging conditions: −0.3 V, 50 pA. Image size: 20 × 20 nm^2^.

In addition to the widespread individual DHC products (Figure 1c), traces of intermolecular coupling products were observed on the Au(111) surface. As marked by the white dashed oval in Figure 4a, two product molecules are morphologically connected with each other via one of their four lobes and proposed to be the intermolecular coupling product. The chemical structure of such species is illustratively superimposed onto an enlarged STM image, which displays well the morphological consistency.

Statistics on the portions of unreacted Co(TAP) molecules, intramolecular DHC products and intermolecular products were further analyzed, aiming at a quantitative understanding of the reaction of the Co(TAP) molecule on Au(111). Before the statistics, the rule for counting each type of molecules is defined as follows. For the DHC reaction, a Co(TAP) molecule either partially or fully turned into the product was counted as one molecule in the statistical process. For intermolecular coupling reaction, whether or not the molecules in an intermolecularly connected chain already underwent DHC reaction, they were counted as intermolecular coupling products only. This ensured that sum of molecular proportions for Co(TAP), intramolecular DHC and intermolecular coupling exactly equaled 100%. For each sample annealed at a different temperature, the STM images collected at different regions of the substrate surface were used for the statistical analyses. In total, 220–250 molecules were counted for each sample. As displayed in Figure 4b, the intramolecular DHC reaction at 523 K involved about 9% of the Co(TAP) molecules. As the annealing temperature increased to 750 K, all Co(TAP) molecules eventually turned into the DHC products, and only a small portion (about 6%) further underwent intermolecular coupling reactions.

According to above-described results, a proposed scenario for the reaction process of the Co(TAP) molecule on Au(111) is as follows: At an elevated temperature of about 523 K, the C–H bonds in the *meso*-phenyl and pyrrole groups can be activated. Additionally, the methoxy groups are about to dissociate as well. However, because the intermolecular Co(TAP) coupling requires the dissociation of the methoxy groups in advance, the intramolecular DHC reaction becomes more feasible. Given that the annealing time is relatively short (2 min), it is proposed that the steric hindrance effect of methoxy group kinetically promotes the selectivity of the intramolecular DHC reaction against the intermolecular counterpart. Once all methoxy groups dissociate, further thermal annealing of the as-prepared sample at 750 K results in the increase of the intermolecular coupling products (Appendix B).

## 3. Materials and Methods

All experiments were performed on a LT-STM (Unisoku USM1300) system with a base pressure lower than 3 × 10^−10^ Torr. An atomically flat Au(111) single crystal (MaTeck GmbH) surface was prepared by cycled argon ion beam bombardment at 2 kV and subsequent high temperature annealing at 750–800 K. The cleanness of the surface was confirmed by the STM imaging; only traces (<3‰) of contaminates (adsorbed H or C atoms) were observed on the Au(111) surface. If present, the contaminating H or C atoms normally appeared as dark depressions in the STM images. Co(TAP) (Sigma-Aldrich, Darmstadt, Germany, 97%) was deposited onto the surface via thermal evaporation from a home-made tantalum boat. The deposition rate of the molecule was monitored with a quartz crystal microbalance (Inficon SQM-160) and kept at 1–2 ML/min during the deposition. Thermal annealing of the sample was conducted by radiation heating at the back of the single crystal. The sample temperature was measured with an infrared thermometer which was calibrated by a thermocouple in advance. The used STM tips were mechanically sheared Pt/Ir alloy wires. All STM and STS measurements were carried out at liquid helium temperature (~4.4 K). All STM images were raw images directly exported from the Rev9 software by RHK Technology. The STS signals were recorded at constant tip height using a lock-in amplifier (SRS SR830) with a bias modulation of V_rms_ = 10 mV at 1720 Hz. The STS data were processed with the Origin software.

The DFT calculations were performed with the Gaussian 09 software (Revision A.02, Wallingford, CT, USA) [31], using the B3LYP functional. For the Co(TAP) molecule and the DHC product, the geometrical optimization was performed by using a combined 6–31 g (d, p) and LANDL2DL basis set. The HOMO and LUMO analyses were based on the correspondingly optimized models.

## 4. Conclusions

In summary, we employed LT-STM and DFT calculations to explore the DHC and intermolecular coupling reactions of Co(TAP) on Au(111). Both STS measurements and DFT calculations strongly supported the proposed chemical structures of the DHC products. At 523 K, the DHC reaction carried on with the dissociation of the methoxy groups. Rapid annealing at higher temperatures led to selective formations of intramolecular DHC products over intermolecular coupling products, implying that the substitution of a protective group such as methoxy could kinetically mediate the selectivity of intra and inter-molecular reactions.

## Figures and Tables

**Figure A1:**
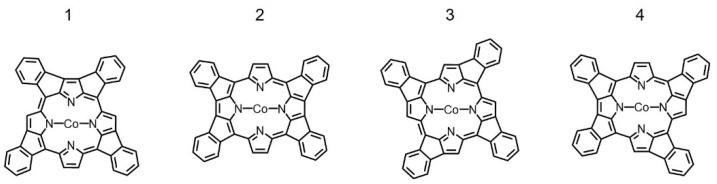
Four structural isomers of the DHC products of Co(TAP) on Au(111).

**Figure A2:**
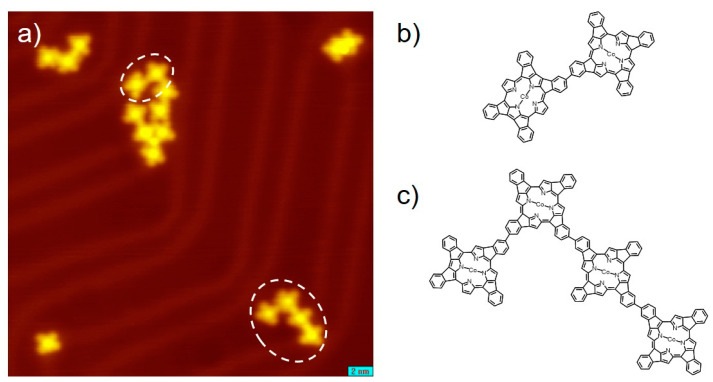
(**a**) STM image of the sample surface of Figure 1c further annealed at about 750 K; (**b**,**c**) proposed chemical structures for two intermolecular coupling products marked by the white dashed ovals in (**a**). Imaging conditions: −0.3 V, 20 pA. Image size: 30 × 30 nm^2^.

**Figure A4:**
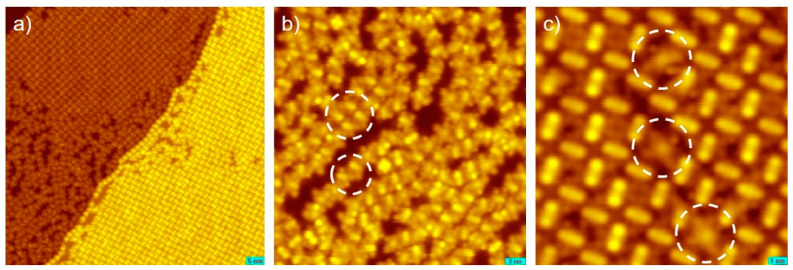
(**a**) Large-scale STM image of the Co(TAP) molecules adsorbed on Au(111) at high coverage and after being annealed at 523 K. (**b**) Enlarged STM images of a disordered domain in (**a**). (**c**) Enlarged STM images of a well-ordered self-assembly domain in (**a**). The intermediate DHC products are marked by the white dashed circles in (**b**,**c**). The blue scale bars in (**a**–**c**) correspondingly represent 5, 2 and 1 nm. Imaging conditions: (**a**,**b**) −1.0 V, 30 pA; (**c**) −0.3 V, 10 pA.

## Appendix A

As the *meso*-phenyl group may react with the pyrrole group on either its left or right side, there exist in total four types of structural isomers for the DHC products, as shown in Figure A1. This rationalizes that the DHC products observed in STM were not uniformly alike. The STM image in inset of Figure 1c represents type 1 isomer. Other isomers were also identifiable according to their imaged morphological shapes and symmetric features. For example, isomers 1 and 2 are mirror symmetric, isomer 3 is centrosymmetric, and isomer 4 is asymmetric. The chemical structure of each molecule in the intermolecular coupling product (enlarged image in Figure 4a) is proposed according to such shapes and symmetric features as well.

## Appendix B

Further annealing of the as-achieved DHC products leads to the increase of intermolecular products (Figure A2a). This is because that most methoxy groups dissociated along with the formations of intramolecular DHC products. After all methoxy groups dissociated, the *para*-H on the *meso*-phenyl group reengaged in the intermolecular reaction to form coupling products. Similar intermolecular coupling products were also previously reported by others [20,21,32]. The chemical structures of two typical intermolecular products (marked by the white dashed ovals in Figure A2a) are proposed in Figure A2b,c, respectively. In addition, essentially no ether-type coupling products were observed according to the STM-imaged morphologies of intermolecular coupling products. This excludes the possible partial dissociation of the methoxy group that would lead to the remaining hydroxy groups.

## Appendix D

The coverage dependence of the DHC reaction of Co(TAP) at elevated temperatures was further investigated. Figure A4a shows the STM image of Co(TAP)-covered Au(111) surface at high molecular coverage after thermal annealing at 523 K. In most of the surface area, the well-ordered self-assembly structures of Co(TAP) [28] remain, while some small domains become disordered. An enlarged STM image of a disordered domain is shown in Figure A4b. Although the image shows a quite messy feature, some molecules, as marked by the white dashed circle, are similar to the intermediate DHC products shown in Figure 3c. Half of the bright rod-like protrusions in Co(TAP) become dim in the STM images of both Figure 3c and Figure A4b. In addition, some molecules in the well-ordered self-assembly domains underwent the DHC reaction. As shown in Figure A4c, the intermediate DHC products similar to those observed in Figure 3b are marked by the dashed circles. According to the STM observations, the DHC reaction of Co(TAP) on Au(111) is not seriously affected by the molecular coverage. Meanwhile, there is no experimental evidence that a high molecular coverage would facilitate the intermolecular coupling between Co(TAP) molecules.
